# Evaluating the impact of intracerebroventricular norepinephrine on spatial memory in rats: Insights into sporadic Alzheimer’s pathogenesis

**DOI:** 10.1016/j.ibneur.2025.12.012

**Published:** 2025-12-22

**Authors:** Mohammad Amir Sharifi Moien, Seyed Javad Saghravanian, Masoud Fereidoni

**Affiliations:** Department of Biology, Faculty of Science, Ferdowsi University of Mashhad, Mashhad, Iran

**Keywords:** Norepinephrine, Morris water maze, Sporadic Alzheimer's, Rats, Streptozotocin

## Abstract

One consequence of stress is the increased release of norepinephrine (NE) in the central nervous system, primarily driven by activation of the sympathetic nervous system. Given the importance of chronic stress in the development and progression of Alzheimer’s disease (AD), clarifying the specific contributions of stress-related pathways, including the sympathetic axis and the hypothalamic-pituitary-adrenal (HPA) axis, is critical. In this study, we examined the effects of repeated central NE administration, as a potential contributor to stress-related cognitive impairment, on spatial memory in rats, alone or in combination with a low-dose streptozotocin (STZ) model of sporadic AD. Forty-nine rats were assigned to seven groups: control (no treatment), sham (saline; i.c.v.), low-dose streptozotocin (0.5 mg/kg, i.c.v.), norepinephrine administration at either 1 (adolescent) or 3 (adult) months of age (30 or 50 μg, respectively; i.c.v.), and co-administration of norepinephrine with streptozotocin at 1 or 3 months of age. Spatial memory was assessed using the Morris Water Maze test. Norepinephrine administration during adolescence and adulthood impaired spatial memory similar to streptozotocin in different parameters of the MWM, with adult rats showing the most significant vulnerability (p < 0.001). However, co-administration of both substances did not exacerbate the impairment caused by each alone. The results suggest that norepinephrine may impair cognition through mechanisms distinct from those of STZ-induced deficits. Additionally, they raise questions about the contribution of the sympathetic axis of chronic stress to the progression of sporadic AD.

## Introduction

Alzheimer’s disease, the most common cause of dementia in old age, is a neurodegenerative condition characterized by memory loss and cognitive impairment (Calabrò et al., 2021). The disease begins with a decline in the ability to retain information, particularly short-term memory, during aging (Tarawneh and Holtzman, 2012). It gradually progresses to loss of temporal orientation (El Haj and Kapogiannis, 2016), depression (Chi et al., 2014), speech impairment (Klimova et al., 2015), social withdrawal (Ike et al., 2020), and ultimately death (Tahami Monfared et al., 2022). Aging is the most significant risk factor for this disease, with the likelihood of developing Alzheimer’s increasing substantially after the age of 65 (Seshadri et al., 1997, Tarawneh and Holtzman, 2012). Besides aging, other factors contribute to the increased risk of Alzheimer’s disease, including stress (Justice, 2018, Caruso et al., 2019).

Stress serves as an environmental, physical, and physiological stimulus capable of disrupting the homeostasis of living organisms (Ross and Van Bockstaele, 2021, Antila et al., 2022). It disrupts learning and memory, primarily through the effects of glucocorticoids (Dorey et al., 2012, Tatomir et al., 2014, Atsak et al., 2016). Clinical studies indicate the detrimental role of glucocorticoids in Alzheimer's disease (Canet et al., 2018, Ouanes and Popp, 2019). Genetic studies also suggest a link between glucocorticoid function and the risk of developing Alzheimer's disease (de Quervain et al., 2004, van Rossum et al., 2008, Lesuis et al., 2018). Numerous pieces of evidence point to hypothalamic-pituitary-adrenal (HPA) axis dysfunction due to its impaired regulatory feedback in the early stages of Alzheimer's disease (Lanté et al., 2015). For example, elevated plasma cortisol levels have been identified in Alzheimer's patients (Ouanes and Popp, 2019, Udeh-Momoh et al., 2019). Furthermore, evidence from animal studies shows an interaction between glucocorticoids and Alzheimer's pathology, including amyloid precursor protein (APP) processing and tau aggregation (Green et al., 2006; Wang et al., 2011). These studies showed that increased glucocorticoid secretion, which is observed at elevated levels in Alzheimer's patients, promotes amyloid-beta formation and tau aggregation following stress induction in animal models of Alzheimer's disease (Carroll et al., 2011, Sotiropoulos et al., 2011, Justice et al., 2015). Moreover, during stress, noradrenergic neurons, particularly those in the locus coeruleus, become highly active, modulating a wide range of brain regions through both excitatory and inhibitory effects (Morilak et al., 2005, Schwarz and Luo, 2015, Mather et al., 2016). However, the specific role of norepinephrine (NE), as a key mediator of chronic stress via sympathetic axis activation, in contributing to cognitive impairment, either in healthy individuals or in animal models of sporadic Alzheimer's disease, remains poorly understood (Ávila-Villanueva et al., 2020).

This study aims to investigate the role of sustained NE signaling, one of the core neurochemical features of the stress response, on the development and severity of cognitive impairments associated with sporadic Alzheimer’s disease in adult male rats. Specifically, the research examines how intermittent intracerebroventricular (i.c.v.) NE injections affect spatial memory performance. Moreover, evaluates the progression of cognitive symptoms in an Alzheimer's disease model in laboratory rats induced by streptozotocin (STZ) (Chen et al., 2014, Gáspár et al., 2021). Over two weeks, intermittent i.c.v. injections of NE are administered to adult male rats. The predominantly stimulatory effects of NE may induce inflammatory changes in the prefrontal cortex and hippocampus, potentially leading to spatial memory deficits (Wang et al., 2021). Additionally, we hypothesized that chronic NE administration may exacerbate Alzheimer 's-like symptoms caused by STZ and possibly lead to further spatial memory deterioration in a mild Alzheimer 's-like model induced by a low dose of i.c.v. STZ in adult male rats (Rostami et al., 2017).

To address these questions and test hypotheses, this study involves chronic administration of NE via i.c.v. in healthy animals as well as in mild Alzheimer 's-like model animals induced by STZ, with spatial memory being assessed using the Morris Water Maze.

## Methods

### Animals

This experimental study was conducted on male Wistar rats, approximately 3 months old (weighing 220–250 g) and 1 month old (weighing 50–70 g). The animals were housed in the animal facility of the Department of Biology, Faculty of Science, Ferdowsi University of Mashhad, under standard conditions: a 12-hour light/dark cycle, a temperature of 22 ± 2°C, humidity of 40–50 %, and no noise pollution. Rats were kept in groups of 5–6 in Plexiglas cages. All breeding, housing, and experimental procedures were conducted following the National Institutes of Health’s *Guide for the Care and Use of Laboratory Animals* (Council, 2011). The ethical procedures in this study followed our previous works published in Rostami et al., 2017, Rostami et al., 2020 and were approved by the Ethics Committee of the Department of Biology at Ferdowsi University of Mashhad (Approval No. 3/41279; Date: 2016–07–12) and carried out in the university’s designated animal facility.

### Experimental groups

**Fig. 1:**
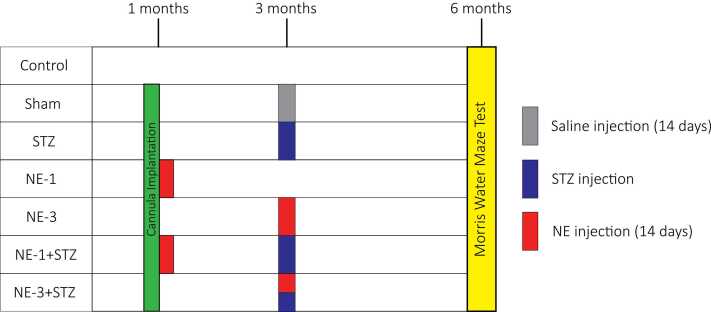
Schematic diagram summarizing the experimental design and treatment groups.

Forty-nine rats were divided into seven groups, each consisting of seven animals. 1. Control group with no treatment. 2. Sham group for evaluating the effects of surgery and solvent injection on memory. 3. STZ group, received i.c.v. injection of 0.5 mg/kg streptozotocin (STZ; Sigma-Aldrich, USA) at 3 months of age to induce an Alzheimer’s-like memory impairment. 4. NE-1 group which received i.c.v. injection of 30 μg norepinephrine (NE; Normon, Spain) in a volume of 5 μl daily for 14 days, starting at 1 month of age, to evaluate the isolated effects of NE on learning. 5. NE-3 group which received i.c.v. injection of 50 μg NE in a volume of 5 μl daily for 14 days, starting at 3 months of age. 6. NE-1_STZ group which received i.c.v. Injection of 30 μg NE in a volume of 5 μl daily for 14 days, starting at 1 month of age, and a single dose of 0.5 mg/kg STZ at 3 months of age. This group was designed to evaluate the potential influence of early-life NE exposure on cognitive outcomes in combination with an Alzheimer’s-like model induced by STZ. 7. NE3_STZ group, which received i.c.v. injection of 50 μg norepinephrine in a volume of 5 μl daily for 14 days and a single dose of 0.5 mg/kg STZ, both starting at 3 months of age (Fig. 1).

The NE3 and NE3-STZ groups included six animals in the statistical analysis due to data loss from one animal in each group.

### Cannula implantation and i.c.v. administration

The animals were anesthetized for cannulation surgery using intraperitoneal ketamine (75 mg/kg) and xylazine (10 mg/kg) (Alfasan, Netherlands). The rats' heads were shaved and fixed in a stereotaxic apparatus (Narishige, Japan). Then, after determining stereotaxic coordinates based on the Paxinos atlas (Paxinos and Watson, 2014), the skull over the right lateral ventricles (0.8 mm posterior to bregma, 1.5 mm lateral to the sagittal suture) was drilled. A 22-gauge stainless steel cannula was implanted (the depth for the cannula tip was set at 3.6 mm beneath the brain surface) and fixed with dental cement. Cannula placement was qualitatively verified in a representative animal by injecting methylene blue dye into the right lateral ventricle. After brain dissection, dye distribution within the lateral ventricles was visually inspected to confirm intraventricular delivery (Ozdemir-Kumral et al., 2024). Systematic histological verification across all animals was not conducted; therefore, the proportion of animals with confirmed ventricular targeting was not quantified. All surgeries were performed using standardized stereotaxic coordinates with individual skull measurements, and no animals were excluded based on cannula placement. Accordingly, this verification served to confirm overall procedural accuracy rather than individual-level validation. At 3 months of age, mild Alzheimer's-like models were induced by injecting 0.5 mg/kg STZ (Rostami et al., 2017) using a 10-μl Hamilton syringe. One week after surgery, groups 4, 5, 6, and 7 received daily NE injections (dosage based on group allocation) for two weeks. All i.c.v. injections were administered at a rate of 5 µl/minute.

The initial dose selection for i.c.v. administration of NE was based on previous studies reporting behavioral and physiological effects of central NE injection in rodents and chicks (Mishra et al., 1993, Zhang et al., 2003). For example, acute doses ranging from 10 to 100 μg have been shown to modulate locomotor activity and arousal, while doses ≥ 200 μg induce immobility and depressive-like behavior (Herman, 1970). Based on these findings, an initial dose of 100 μg NE was tested in pilot trials. However, mortality was observed within two days of injection. The dose was then reduced to 75 μg, yet further mortality occurred. Ultimately, a dose of 50 μg was identified as the highest tolerable dose that maintained animal survival over the 14-day injection period and was thus used for adult rats (NE-3 group). For the NE-1 group (injected at 1 month of age), the dose was further reduced to 30 μg to account for the smaller brain of juvenile rats. This adjustment aimed to avoid excessive central NE concentrations that could lead to non-specific toxicity or stress-unrelated pathology.

Note that the sham group animals underwent the same surgical procedure but received an equivalent volume of saline instead of STZ or norepinephrine.

### Morris water maze

To evaluate cognitive changes, particularly in spatial learning and memory, in the STZ-induced Alzheimer-like model and to assess the effects of norepinephrine, the Morris Water Maze (MWM) was utilized (R. Morris, 1984). The MVM test followed the protocol reported in the previous study, with some modifications (Rostami et al., 2017). In summary. this apparatus consists of a cylindrical black pool with an inner diameter of 150 cm and a height of 80 cm, filled with water maintained at a temperature of 23 ± 1°C to a depth of 25 cm. A small transparent Plexiglas platform with a diameter of 10 cm was placed 1 cm below the water surface in the center of one quadrant (the northeast quadrant, designated as quadrant 1). Rats were randomly released into the pool from one of the four quadrants, and the time taken to locate the platform and the distance traveled were recorded and analyzed using ANY-maze Video Tracking Software (ANY-maze, Stoelting Co., IL, USA). Each rat underwent training for five consecutive days, with one session per day. Each session consisted of four trials, starting randomly from one of the four directions of the pool. A trial ended either when the rat located the platform or after 60 s. After each trial, the rat was allowed to remain on the platform for 20 s before the next trial began. Rats that failed to locate the platform within 60 s were guided to it by the experimenter and allowed to stay on it for 15 s. After completing the fourth trial, the rats were removed from the pool. On the sixth day, the platform was removed, and a probe trial was conducted to assess the animal's recall of the platform's location from previous training. The data collected included the distance traveled and the duration of time spent in each of the four quadrants during the probe trial. The MWM was employed in this study as a reliable method to assess spatial memory, as the tasks involved in this system require proper memory function (Anacker et al., 2011, Vorhees and Williams, 2006). All experimental groups underwent the MWM test at the age of 6 months.

### Data analysis

Following each session, the average latency (or time spent in the target quadrant for the probe session; in seconds) and distance traveled (or distance searched in the target quadrant for the probe session; in meters) to reach the platform across four trials were calculated. The average search speed (m/s) for the session was then determined using the formula:

$$\mathrm{SS}=\frac{\mathrm{DT}}{L}$$

where *SS* represents the search speed, *DT* is the average distance traveled (distance searched in the target quadrant for the probe session) in meters, and *L* is the average latency (time spent in the target quadrant for the probe session) in seconds.

To assess within-group changes over time, a repeated-measures one-way ANOVA was performed. Comparisons between groups and across sessions were evaluated using a two-way ANOVA followed by Bonferroni post hoc tests. For the probe session, a one-way ANOVA followed by Bonferroni post hoc tests was conducted to compare results between groups. A p-value of < 0.05 was considered statistically significant. All statistical analyses and graphical representations were performed using MATLAB (The MathWorks Inc., Natick, MA).

## Result

**Fig. 2:**
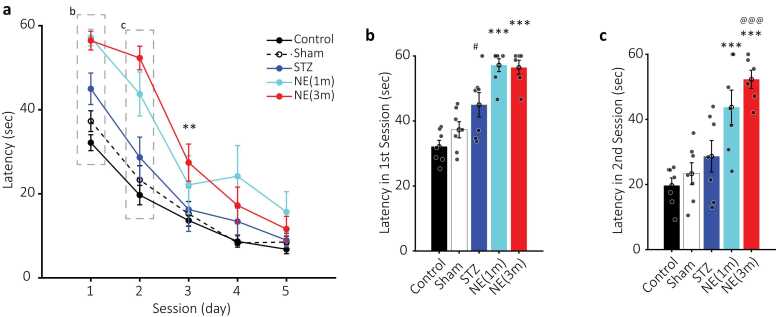
Mean latency of experimental groups in the Morris Water Maze (MWM). a) Mean latency (seconds) for control (black circles), sham (empty black circles, dashed line), STZ (blue circles), NE1 (cyan circles), and NE3 (red circles) across five consecutive training days. Dashed rectangles highlight data presented in b and c. Statistical significance is indicated by ** (p < 0.01) for NE3 vs. control. b-c) Bar plots of mean latency for the first and second sessions, respectively. Colors match those in a. Statistical significance is denoted by *** (p < 0.001) for NE groups vs. control. @@@ (p < 0.001) indicates significant differences between NE and STZ groups, while # (p < 0.05) marks differences between STZ and control. Data are presented as mean ± SEM, n = 7 per group.

**Fig. 3:**
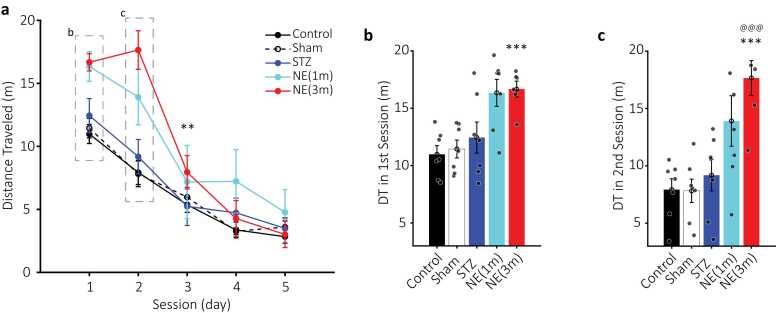
Distance traveled (DT) in the MWM. a) Plots of average DT (meters) for control (black circles), sham (empty black circles, dashed line), STZ (blue circles), NE1 (cyan circles), and NE3 (red circles) over five training days. Dashed rectangles highlight data shown in b and c. ** (p < 0.01) indicates significant differences between NE3 and control. b-c) Bar plots of DT for the first and second sessions. Colors match those in a. Statistical significance is indicated by *** (p < 0.001) for NE3 vs. control and @@@ (p < 0.001) for NE3 vs. STZ. Data are shown as mean ± SEM, n = 7 per group.

**Fig. 4:**
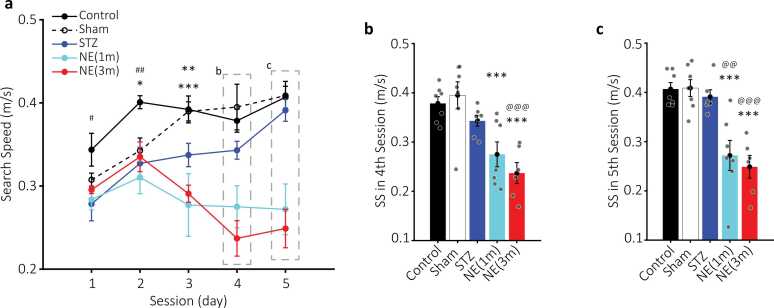
Search speed (SS) of the MWM experimental groups. a) Plots of average SS (meters/second) for control (black circles), sham (empty black circles, dashed line), STZ (blue circles), NE1 (cyan circles), and NE3 (red circles) across five training days. Dashed rectangles highlight data presented in b and c. Statistical significance: * (p < 0.05) for NE1 vs. control, ** (p < 0.01), and *** (p < 0.001) for NE3 vs. control. # (p < 0.05) and ## (p < 0.01) indicate differences between STZ and control. b-c) Bar plots of SS for the fourth and fifth sessions. Colors match those in a. *** (p < 0.001) denotes differences between NE groups and control. @@ (p < 0.01) and @@@ (p < 0.001) indicate significant differences between NE and STZ groups. Data are mean ± SEM, n = 7 per group.

**Fig. 5:**
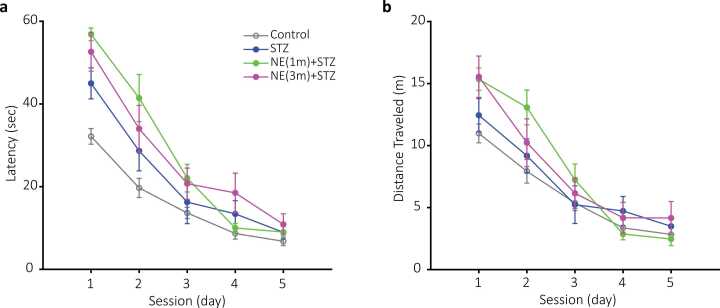
Latency and distance traveled (DT) in co-administration groups. a) Mean latency (seconds) for STZ (blue circles), NE1-STZ (green circles), and NE3-STZ (magenta circles) over five training days. b) Average DT (meters) for the same groups. No significant differences were detected between groups in either latency or DT across sessions. The control group is included in gray for reference. Data are presented as mean ± SEM, n = 7 per group.

**Fig. 6:**
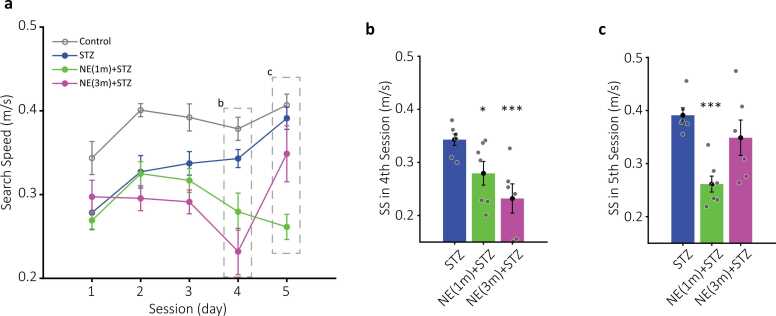
Search speed (SS) in co-administration groups. a) Plots of average SS (meters/second) for STZ (blue circles), NE1-STZ (green circles), and NE3-STZ (magenta circles) over five training days. Dashed rectangles highlight data displayed in b and c. The control group is included in gray for reference. b-c) Bar plots of SS for the fourth and fifth sessions. Colors match those in a. Statistical significance: * (p < 0.05) and *** (p < 0.001) for comparisons between NE-STZ and STZ groups. Data are presented as mean ± SEM, n = 7 per group.

**Fig. 7:**
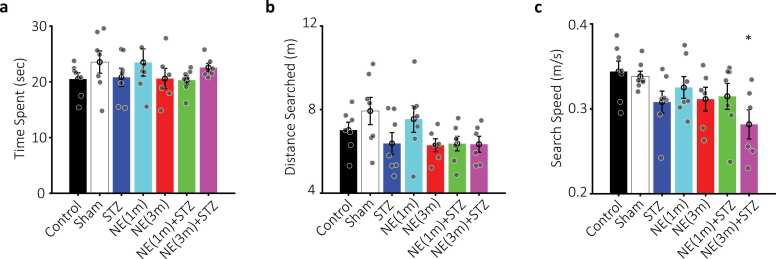
Probe session performance in the MWM. Bar plots of a) average time spent (seconds), b) distance searched (meters), and c) search speed (meters/second) during the probe session across groups in the target quadrant, previously associated with the platform location. Colors match previous figures. * (p < 0.05) denotes significant differences between NE3-STZ and control. Data are shown as mean ± SEM, n = 7 per group.

In this study, we evaluated the effect of intracerebroventricular (i.c.v.) injection of norepinephrine (NE) on spatial memory in rats using the Morris Water Maze (MWM) test. To better assess the impact of NE, a low dose of streptozotocin (STZ), a well-established compound for inducing sporadic Alzheimer’s disease in rats (Chen et al., 2014, Gáspár et al., 2021), was also administered in some groups. A total of 49 rats were divided into seven groups. Five groups, including 1. Control (no treatment; black lines and bars in Fig. 2, Fig. 3, Fig. 4, Fig. 7), 2. Sham (saline-injected; black dashed lines and white bars with black edges in Fig. 2, Fig. 3, Fig. 4, Fig. 6), 3. STZ (STZ injected at 3 months of age; blue lines and bars in Fig. 2, Fig. 3, Fig. 4, Fig. 7), 4. NE-1 (NE injected at 1 month of age; cyan lines and bars in Fig. 2, Fig. 3, Fig. 4, Fig. 7), 5. NE-3 (NE injected at 3 months of age; red lines and bars in Fig. 2, Fig. 3, Fig. 4, Fig. 7). Moreover, two groups were to evaluate the combined effects of NE and low-dose STZ, including 6. NE-1_STZ (NE injected at 1 month of age and STZ at 3 months of age; green lines and bars in Fig. 5, Fig. 6, Fig. 7), and 7. NE-3_STZ (NE and STZ injected at 3 months of age; magenta lines and bars in Fig. 5, Fig. 6, Fig. 7). All groups underwent a five-day training protocol in the MWM, and their performance was assessed based on (a) latency to find the platform, (b) distance traveled (DT) to reach the platform, and (c) search speed (SS) across each session (day). The sixth day was introduced as the probe in which the position of the platform changed, and all the above parameters at the target quadrant (past location of the platform) were evaluated.

### Norepinephrine (NE) impact on latency

Both NE groups increased the latency to reach the platform compared to the control group (two-way ANOVA; NE1: F (1, 12) = 11.6, p < 0.01; NE3: F (1, 11) = 41.4, p < 0.001; Fig. 2a). The increased latency was more pronounced during the 1st (control= 32.1 ± 1.8; NE1 = 57.1 ± 1.9; NE3 = 56.4 ± 2.2 s; Fig. 2b) and 2nd (control= 16.6 ± 2.3; NE1 = 52.3 ± 2.8; NE3 = 43.7 ± 5.2 s; Fig. 2c) sessions of the NE1 and NE3 groups (Bonferroni; p < 0.001). Moreover, the 3rd session of the NE3 group had longer latency compared to the control group (control = 13.6 ± 1.3; NE3 = 27.4 ± 4.4 s; Bonferroni; p < 0.01; Fig. 2a).

The low dose of STZ itself did not increase the latency during 5 days of training compared to the control (two-way ANOVA; F (1, 12) = 3.22, p = 0.09; Fig. 2a). However, the latency during the 1st session reached significance (control= 32.1 ± 1.8; STZ= 44.9 ± 3.7 s; Bonferroni; p < 0.05; Fig. 2b). Although NE groups had greater latency relative to the STZ, only the NE3 significantly differed from it (two-way ANOVA; NE1: F (1, 12) = 3.24, p = 0.09; NE3: F (1, 11) = 6.2, p < 0.05; Fig. 2a). The latency difference was statistically significant during the 2nd session of the NE3 compared to the STZ group (NE3 = 43.7 ± 5.2; STZ= 28.6 ± 4.8 s; Bonferroni; p < 0.001; Fig. 2c).

### Norepinephrine (NE) impact on distance traveled (DT)

Only the NE3 group significantly increased DT to reach the platform compared to the control group (two-way ANOVA; F (1, 11) = 15.7, p < 0.01; Fig. 3a). The increased DT was more pronounced during the 1st (control= 10.9 ± 0.75; NE3 = 16.6 ± 0.68 m; Fig. 3b) and 2nd (control= 7.9 ± 0.95; NE3 = 17.6 ± 1.5 m; Fig. 3c) sessions of the NE3 group (Bonferroni; p < 0.001). Moreover, the 3rd session of the NE3 group had a longer DT compared to the control group (Bonferroni; p < 0.01; Fig. 3a). However, the NE1 group also showed an increase in overall DT (1st = 16.3 ± 1.1; 2nd = 13.9 ± 2.2 m; Fig. 3) but did not reach significance compared to the control group (two-way ANOVA; F (1, 12) = 3.8, p = 0.07).

Similar to latency, the STZ group didn’t change the DT compared to the control group (two-way ANOVA; F (1, 12) = 0.65, p = 0.43; Fig. 3a). Two-way ANOVA showed no significant difference between the NE groups and STZ in DT (NE1: F (1, 12) = 1.86, p = 0.19; NE3: F (1, 11) = 4.52, p < 0.057; Fig. 3a). However, NE3 during the 2nd session (STZ= 9.1 ± 1.3; NE3 = 17.6 ± 1.5 m; Fig. 3c) significantly increased DT compared to the STZ group (Bonferroni; p < 0.001).

### Norepinephrine (NE) impact on average search speed (SS)

The SS results showed a positive trend toward faster search among the control (0.3 ± 0.01–0.4 ± 0.01 m/s during five training sessions), sham (0.3 ± 0.007–0.4 ± 0.01 m/s), and STZ (0.27 ± 0.02–0.39 ± 0.01 m/s) groups after training (repeated-measures one-way ANOVA; control: F (2.895, 17.37) = 3.287, p < 0.05; sham: F (1.804, 10.82) = 9.806, p < 0.01; STZ: F (2.484, 14.28) = 7.549, p < 0.01; Fig. 4). However, the positive trend in SS following training tended to suppress or reverse toward slower speeds in NE1 (0.28 ± 0.01–0.27 ± 0.03 m/s) and NE3 (0.29 ± 0.004–0.24 ± 0.02 m/s) groups, respectively (repeated-measures one-way ANOVA; NE1: F (2.333, 14.00) = 0.5935, p = 0.59; NE3: F (2.314, 11.57) = 5.757, p < 0.01; Fig. 4a).

NE groups significantly decreased SS compared to the control group (two-way ANOVA; NE1: F (1, 12) = 23, p < 0.001; NE3: F (1, 11) = 79.2, p < 0.001; Fig. 4a). The slower SS in NE groups was more pronounced during the last four sessions compared to the control group (Bonferroni; NE1: 1st p = 0.26; 2nd p < 0.05; 3rd p < 0.01; 4th p < 0.01; 5th p < 0.001; NE3: 1st p = 0.17; 2nd p < 0.05; 3rd to 5th p < 0.001; Fig. 4a-c).

The STZ group had slower SS in the first 2 sessions compared to the control group, but became more similar to the control group following the training (two-way ANOVA; F (1, 12) = 15.2, p < 0.01; Bonferroni; 1st p < 0.05 and 2nd p < 0.01; Fig. 4a). Furthermore, NE groups had slower SS relative to the STZ (two-way ANOVA; NE1: F (1, 12) = 5.79, p < 0.05; NE3: F (1, 11) = 17.5, p < 0.01). The SS difference was statistically significant during the 5th session of the NE1 (Bonferroni; p < 0.01; Fig. 4c) and the 4th and 5th sessions of the NE3 groups (Bonferroni; p < 0.001; Fig. 4b&c) compared to the STZ group.

There was no significant difference in latency, distance traveled, or search speed between the sham and control groups during different sessions, which shows that the surgical procedure and i.c.v. injection did not impact spatial memory itself (two-way ANOVA; latency: F (1, 12) = 2.95, p = 0.11; distance traveled: F (1, 12) = 0.26, p = 0.61; search speed: F (1, 12) = 1.17, p = 0.3; Fig. 4).

### Effects of NE and STZ Co-administration on spatial memory

During the study, we evaluated the combination effect of NE and STZ during two different exposure times of NE (one or three months of age; see method section) and compared it to the STZ group. NE_STZ groups tend to increase both latency and DT in the first two sessions, However, none of them reached significance compared to the STZ group (two-way ANOVA; for latency − NE1_STZ: F (1, 12) = 2.25, p = 0.15; NE3_STZ: F (1, 11) = 1.36, p = 0.26; for DT − NE1_STZ: F (1, 12) = 1.2, p = 0.29; NE3_STZ: F (1, 11) = 0.54, p = 0.47; Fig. 5). Moreover, none of the NE-STZ groups had substantial difference with their respective NE alone group (two-way ANOVA; for latency − NE1_STZ: F (1, 12) = 0.85, p = 0.37; NE3_STZ: F (1, 10) = 2.1, p = 0.17; for DT − NE1_STZ: F (1, 12) = 0.72, p = 0.41; NE3_STZ: F (1, 10) = 1.64, p = 0.22; Fig. 5).

Average search speed of the NE_STZ groups were similar to NE alone groups (two-way ANOVA; NE1_STZ: F (1, 12) = 0.1, p = 0.75; NE3_STZ: F (1, 10) = 0.5, p = 0.49). Both groups showed lower speed compared to the STZ alone group following the training (two-way ANOVA; NE1_STZ: F (1, 12) = 14.3, p < 0.01; NE3_STZ: F (1, 11) = 6.78, p < 0.05; Fig. 6a). The difference was more pronounced during the 4th (NE1_STZ= 0.27 ± 0.02; STZ= 0.34 ± 0.01 m/s; Fig. 6b) and 5th (NE1_STZ= 0.26 ± 0.01; STZ= 0.39 ± 0.01 m/s; Fig. 6c) sessions of the NE1_STZ compared to the STZ group (Bonferroni; 4th p < 0.05; 5th p < 0.001). However, only the 4th session (0.23 ± 0.02 m/s; Fig. 6b) of the NE3_STZ reached a statistically significant difference compared to the STZ group (Bonferroni; p < 0.001).

### Probe session

Fig. 7 illustrates the results of the probe session. In this session, the platform’s position was changed to quantify the animals' search parameters in the target quadrant, previously associated with the platform location. Statistical analysis revealed no significant differences among groups during the probe session for time spent (TS) or distance searched (DS) in the target quadrant (one-way ANOVA; TS: F (6, 40) = 0.79, p = 0.57; DS: F (6, 40) = 1.89, p = 0.1). However, a significant group effect was observed for search speed (SS) (F(6, 40) = 2.42, p = 0.04; Fig. 7c), with post hoc analysis showing that the NE3-STZ group exhibited significantly slower SS compared to the control group (Bonferroni; p < 0.05).

## Discussion

This study aimed to examine whether sustained central norepinephrine (NE) exposure, a key feature of the neurochemical stress response, contributes to Alzheimer’s-like spatial memory deficits in male rats. To this end, we administered repeated intracerebroventricular (i.c.v.) NE injections and assessed their impact on spatial memory performance—a cognitive domain commonly disrupted in Alzheimer’s disease (Jacobsen et al., 2006, Zhu et al., 2017). The Morris Water Maze (MWM) was employed as a standard method to assess spatial memory performance in rats (Morris, 1984; Vorhees and Williams, 2006). NE was i.c.v. administered daily for two weeks at two different developmental stages (Spear, 2000). Some groups (NE1 and NE1_STZ) received NE at one month of age, representing early-life exposure during adolescence, while others (NE3 and NE3_STZ) received it at three months of age, corresponding to adulthood. Additionally, a low dose of streptozotocin (STZ; 0.5 mg/kg) was administered alone or in combination with NE to evaluate the potential exacerbation of spatial memory impairment by NE. The low STZ dose was selected to induce mild cognitive impairment (MCI) in spatial memory (Li et al., 2016, Rostami et al., 2017, Rostami et al., 2020), allowing for an assessment of the combined effects of NE (as a chronic stress factor) in an MCI model.

Results showed that NE administration during both adolescence and adulthood impaired spatial memory in the Morris Water Maze (MWM). This impairment was significantly greater in the NE3 group compared to the control group during the first three sessions, as reflected in key performance measures, including latency and distance traveled to reach the platform (Fig. 2, Fig. 3). Similarly, the NE1 group exhibited a comparable impairment pattern, though statistical significance was reached only for increased latency during the first two sessions (Fig. 2). Additionally, the NE3 group demonstrated a significant increase in latency and distance traveled compared to the STZ group in the second session (Fig. 2, Fig. 3). NE administration also disrupted the typical increase in search speed (SS) observed across sessions in the control, sham, and even STZ groups (Fig. 4). This disruption was statistically significant in the last three sessions when comparing the NE1 and NE3 groups to the control group (Fig. 4). Furthermore, the NE3 group exhibited slower SS in the fourth and fifth sessions compared to the STZ group (Fig. 4b&c). The NE1 group followed a similar trend, though a significantly slower SS from the STZ group was only observed in the fifth session (Fig. 4c). Groups that received both NE and STZ exhibited a greater, though not statistically significant, impairment in latency and distance traveled to reach the platform compared to the STZ-alone group (Fig. 5). However, the NE1-STZ group demonstrated significantly slower search speed (SS) in the last two sessions compared to the STZ-alone group. Additionally, the NE3-STZ group showed a significantly slower SS compared to the STZ-alone group during the fourth session (Fig. 6). All the employed results in the discussion were summarized in Table 1.

**Table 1:** Relative changes among experimental groups in MWM. Relative changes between each pair of groups in different parameters based on two-way ANOVA. Relative increase or decrease is shown with upward or downward arrows, respectively.

^1^ not significant; ^2^ p < 0.05; ^3^ p < 0.01; ^4^ p < 0.001.

The neurotoxic effects of STZ on central nervous system neurons and its role in impairing learning and memory have been well documented (Shoham et al., 2003, Chen et al., 2014, Gáspár et al., 2021). When administered intracerebroventricularly, STZ induces neurodegenerative, biochemical, and molecular changes, such as impaired cerebral glucose metabolism, oxidative stress, and neuroinflammation, that closely resemble key features of sporadic Alzheimer’s disease (sAD) (Salkovic-Petrisic et al., 2013, Li et al., 2016). While various doses have been used to model different severities of AD-like pathology, our study employed a low dose of 0.5 mg/kg, consistent with prior reports by Rostami et al., 2017, Rostami et al., 2020. In those studies, this dose was shown to produce mild cognitive deficits alongside validated molecular and histological changes, making it suitable for modeling early-stage sAD or MCI. The aim of using this subthreshold dose was to enable examination of potential additive or modulatory effects of NE administration on a relatively mild pathological background, rather than overwhelming the system with severe neurodegeneration.

Norepinephrine, as a neurotransmitter in the central nervous system primarily released from the locus coeruleus, plays multiple roles, including the activation of the HPA axis (Charmandari et al., 2005; Morris et al., 2020). The repeated activation of the HPA axis leads to an increase in glucocorticoid secretion (Herman et al., 2016), which is generally associated with impairments in learning and memory (Dorey et al., 2012, Tatomir et al., 2014, Atsak et al., 2016). Excessive exposure to glucocorticoids due to increased norepinephrine levels induces adaptive changes in glucocorticoid receptors, particularly in the hippocampus, leading to hippocampal atrophy and reduced neurogenesis (Vyas et al., 2002, Anacker et al., 2011, Finsterwald and Alberini, 2014). Hippocampal damage or atrophy is linked to impaired negative feedback regulation of the HPA axis, resulting in prolonged HPA axis activation and further neuronal damage (Lanté et al., 2015).

The present study showed that exposure to excessive NE during adolescent or adult life stages can induce mild cognitive impairment (MCI) comparable to that produced by a low dose of STZ. Although both NE1 and NE3 groups exhibited similar impairments, the NE3 group showed more pronounced deficits relative to both the control and STZ groups, suggesting that NE exposure during adulthood leads to greater cognitive disruption. This heightened vulnerability in 3-month-old rats may stem from reduced compensatory plasticity compared to younger animals, making them more susceptible to NE-induced damage (Spear, 2013). For example, a study examining the induction of mRNA for tissue plasminogen activator, a marker of neural plasticity, found a delayed and blunted response in 3-month-old rats compared to younger ones (Popa-Wagner et al., 2000). This suggests that the mature brain's reduced capacity for plastic changes may contribute to greater vulnerability to neurochemical insults. Additionally, age-related neuronal loss begins to manifest around 3 months of age in rats, with progressive declines observed in various brain structures (Morterá and Herculano-Houzel, 2012). This neuronal attrition may further compromise the brain's resilience to NE-induced impairments in older rats.

Importantly, although not included in the present manuscript, unpublished histological analyses from our laboratory confirm that chronic NE exposure (same protocol as the current study) leads to structural alterations in the hippocampus, comparable in severity and distribution to those observed in STZ-treated animals. These findings align with the behavioral outcomes reported here and support the view that NE-induced impairments are not merely functional or transient but may reflect underlying neuropathological changes.

We hypothesized that NE contributes to the development of sporadic Alzheimer's induced by STZ. However, co-administration of NE and STZ didn’t result in a greater impairment than the administration of NE or STZ alone. The lack of a cumulative effect in the co-administration of NE and STZ may be due to differences in their underlying mechanisms of action, which could limit their interaction in producing more severe deficits. While NE primarily induces cognitive impairment through HPA axis overactivation and glucocorticoid-related neurotoxicity (Dorey et al., 2012, Tatomir et al., 2014, Atsak et al., 2016), STZ disrupts insulin signaling and glucose metabolism, leading to neuronal dysfunction (Salkovic-Petrisic et al., 2013, Li et al., 2016, Rostami et al., 2017). These mechanisms, although both capable of causing mild cognitive impairment (MCI), may not strongly interact or amplify each other’s effects. Additionally, STZ-induced metabolic dysfunction could have altered NE sensitivity, potentially reducing the impact of additional NE administration (Yoshida et al., 1985). On the other hand, NE administration could excite the brain and enhance neural activity (Jhaveri et al., 2010, Mather et al., 2016). This increased activity, in turn, may promote greater cerebral blood flow, facilitating the clearance of STZ-induced metabolic byproducts (such as amyloid-beta) and increasing brain oxygenation, which are both associated with sporadic Alzheimer's disease (Bjerkan et al., 2024, Meng et al., 2024). Consequently, although both agents individually impaired learning and memory, their co-administration did not lead to a significantly greater cognitive decline than either treatment alone. This study demonstrated that repetitive i.c.v. administration of NE, for mimicry of the action of the sympathetic axis of the repetitive chronic stress on the brain, did not exacerbate the progression of STZ-induced sporadic Alzheimer's disease. Therefore, these findings cast doubt on the involvement of the sympathetic axis in the progression of sporadic Alzheimer's disease and instead highlight the HPA axis as a key contributor to stress-related disease progression.

Results from the NE and STZ groups in the Morris Water Maze (MWM) indicated that mild cognitive impairment (MCI) could be partially mitigated through repeated training, as reflected by reductions in escape latency and distance traveled across sessions. By the later stages of acquisition, the spatial performance of these groups became comparable to that of controls. However, animals in the NE-treated groups, particularly those exhibiting more pronounced initial impairments, demonstrated this improvement alongside a significant reduction in search speed. This pattern suggests that while training facilitated performance, it may have done so by engaging compensatory mechanisms that rely on slower, more deliberate cognitive processing. These findings align with previous evidence indicating that individuals with MCI or subtle learning impairments can improve task accuracy at the cost of efficiency, often reflected in longer decision times or slower navigation (Wadley et al., 2008, Tangen et al., 2015, Lassen-Greene et al., 2017).

Additionally, traditional probe measures did not reveal overt deficits in spatial memory retention. The reduced search speed in NE-treated animals, particularly in the NE3-STZ group, suggests a compensatory change in search strategy. These animals may have adopted a more cautious, less efficient exploration pattern to overcome underlying impairments in spatial learning observed during acquisition. Thus, the disruption appears to affect the learning process more prominently than memory retrieval alone, and repeated training may have partially compensated for these deficits by shifting behavioral strategies.

In conclusion, repeated intracerebroventricular administration of norepinephrine (NE) during different life stages induced mild cognitive deficits in spatial memory, resembling those seen with STZ treatment. However, these impairments likely arise through distinct neurobiological pathways. Interestingly, co-administration of NE and STZ did not exacerbate cognitive decline, suggesting possible compensatory interactions between their mechanisms of action. These findings raise questions about the specific contribution of central NE to Alzheimer’s-like pathology and underscore the need to further investigate alternative stress-related pathways, such as the HPA axis, in the context of sporadic Alzheimer’s disease.

## Future direction

This study focused on evaluating the effects of intracerebroventricular norepinephrine (NE) and streptozotocin (STZ) on spatial memory using the Morris Water Maze. However, our findings remain open to alternative interpretations. NE is known to influence arousal and locomotion, and without additional behavioral tests, such as the open field test or novel object recognition, or a cued version of the Morris Water Maze, it is difficult to fully rule out whether the observed impairments are due to cognitive deficits or other factors such as changes in motivation, vision, or swimming ability. Moreover, we did not measure biochemical markers of hypothalamic-pituitary-adrenal (HPA) axis function, and thus cannot exclude the possibility that alterations in stress hormone levels contributed to the observed effects. Future studies should include a broader behavioral assessment and HPA axis evaluation to clarify these issues.

Additionally, our results suggest that NE and STZ may affect memory through different mechanisms. Future research should examine relevant neurobiological factors, such as hippocampal inflammation, stress hormone levels (e.g., corticosterone), and Alzheimer’s-related pathology (e.g., beta-amyloid or tau), to better understand the specific mechanisms underlying the cognitive effects we observed.

Although intraventricular cannula placement was qualitatively verified in a representative animal using methylene blue dye, systematic histological confirmation was not performed across the entire experimental cohort. Consequently, the exact proportion of animals with accurate ventricular targeting could not be quantified, and injections were not screened on an individual basis. While all surgeries were conducted using standardized stereotaxic coordinates with individual skull measurements and produced consistent behavioral effects within groups, future studies should incorporate comprehensive histological verification in all animals to strengthen methodological rigor and ensure precise anatomical targeting.

## CRediT authorship contribution statement

**Masoud Fereidoni:** Writing – review & editing, Writing – original draft, Validation, Supervision, Software, Resources, Project administration, Methodology, Investigation, Funding acquisition, Formal analysis, Conceptualization. **Mohammad Amir Sharifi Moien:** Writing – original draft, Project administration, Methodology, Investigation, Formal analysis, Data curation, Conceptualization. **Seyed Javad Saghravanian:** Writing – review & editing, Writing – original draft, Visualization, Validation, Software, Formal analysis, Data curation, Conceptualization.

## Conflicts of Interest

The authors declare that they have no known competing financial interests or personal relationships that could have appeared to influence the work reported in this paper.

## References

[bib1] Anacker C., Zunszain P.A., Carvalho L.A., Pariante C.M. (2011). The glucocorticoid receptor: pivot of depression and of antidepressant treatment?. Psychoneuroendocrinology.

[bib2] Antila H., Kwak I., Choi A., Pisciotti A., Covarrubias I., Baik J., Eisch A., Beier K., Thomas S., Weber F., Chung S. (2022). A noradrenergic-hypothalamic neural substrate for stress-induced sleep disturbances. Proc. Natl. Acad. Sci. USA.

[bib3] Atsak P., Guenzel F.M., Kantar-Gok D., Zalachoras I., Yargicoglu P., Meijer O.C., Quirarte G.L., Wolf O.T., Schwabe L., Roozendaal B. (2016). Glucocorticoids mediate stress-induced impairment of retrieval of stimulus-response memory. Psychoneuroendocrinology.

[bib4] Ávila-Villanueva M., Gómez-Ramírez J., Maestú F., Venero C., Ávila J., Fernández-Blázquez M.A. (2020). The role of chronic stress as a trigger for the Alzheimer disease continuum. Front. Aging Neurosci..

[bib5] Bjerkan J., Meglič B., Lancaster G., Kobal J., McClintock P.V.E., Crawford T.J., Stefanovska A. (2024). Neurovascular phase coherence is altered in Alzheimer’s disease. Brain Commun..

[bib6] Calabrò M., Rinaldi C., Santoro G., Crisafulli C. (2021). The biological pathways of Alzheimer disease: a review. AIMS Neurosci..

[bib7] Canet G., Chevallier N., Zussy C., Desrumaux C., Givalois L. (2018). Central role of glucocorticoid receptors in Alzheimer’s disease and depression. Front. Neurosci..

[bib8] Carroll J.C., Iba M., Bangasser D.A., Valentino R.J., James M.J., Brunden K.R., Lee V.M.Y., Trojanowski J.Q. (2011). Chronic stress exacerbates tau pathology, neurodegeneration, and cognitive performance through a corticotropin-releasing factor receptor-dependent mechanism in a transgenic mouse model of tauopathy. J. Neurosci..

[bib9] Caruso A., Nicoletti F., Gaetano A., Scaccianoce S. (2019). Risk factors for Alzheimer’s disease: focus on stress. Front. Pharmacol..

[bib10] Charmandari E., Tsigos C., Chrousos G. (2005). Endocrinology of the stress response. Annu. Rev. Physiol..

[bib11] Chen Y., Liang Z., Tian Z., Blanchard J., Dai C.L., Chalbot S., Iqbal K., Liu F., Gong C.X. (2014). Intracerebroventricular streptozotocin exacerbates alzheimer-like changes of 3xTg-AD mice. Mol. Neurobiol..

[bib12] Chi S., Yu J.T., Tan M.S., Tan L. (2014). Depression in Alzheimer’s disease: epidemiology, mechanisms, and management. J. Alzheimer’S. Dis..

[bib13] Council N.R. (2011).

[bib14] de Quervain D.J.F., Poirier R., Wollmer M.A., Grimaldi L.M.E., Tsolaki M., Streffer J.R., Hock C., Nitsch R.M., Mohajeri M.H., Papassotiropoulos A. (2004). Glucocorticoid-related genetic susceptibility for Alzheimer’s disease. Hum. Mol. Genet..

[bib15] Dorey R., Piérard C., Chauveau F., David V., Béracochéa D. (2012). Stress-induced memory retrieval impairments: different time-course involvement of corticosterone and glucocorticoid receptors in dorsal and ventral hippocampus. Neuropsychopharmacology.

[bib16] El Haj M., Kapogiannis D. (2016). Time distortions in alzheimer’s disease: a systematic review and theoretical integration. npj Aging Mech. Dis..

[bib17] Finsterwald C., Alberini C.M. (2014). Stress and glucocorticoid receptor-dependent mechanisms in long-term memory: from adaptive responses to psychopathologies. Neurobiol. Learn. Mem..

[bib18] Gáspár A., Hutka B., Ernyey A.J., Tajti B.T., Varga B.T., Zádori Z.S., Gyertyán I. (2021). Intracerebroventricularly injected streptozotocin exerts subtle effects on the cognitive performance of long-evans rats. Front. Pharmacol..

[bib19] Green K.N., Billings L.M., Roozendaal B., McGaugh J.L., LaFerla F.M. (2006). Glucocorticoids increase amyloid-β and tau pathology in a mouse model of Alzheimer’s disease. J. Neurosci..

[bib20] Herman J.P., McKlveen J.M., Ghosal S., Kopp B., Wulsin A., Makinson R., Scheimann J., Myers B. (2016). Regulation of the hypothalamic-pituitary- adrenocortical stress response. Compr. Physiol..

[bib21] Herman Z.S. (1970). The effects of noradrenaline on rat’s behaviour. Psychopharmacologia.

[bib22] Ike K.G.O., de Boer S.F., Buwalda B., Kas M.J.H. (2020). Social withdrawal: an initially adaptive behavior that becomes maladaptive when expressed excessively. Neurosci. Biobehav. Rev..

[bib23] Jacobsen J.S., Wu C.C., Redwine J.M., Comery T.A., Arias R., Bowlby M., Martone R., Morrison J.H., Pangalos M.M., Reinhart P.H., Bloom F.E. (2006). Early-onset behavioral and synaptic deficits in a mouse model of Alzheimer’s disease. Proc. Natl. Acad. Sci. USA.

[bib24] Jhaveri D.J., Mackay E.W., Hamlin A.S., Marathe S.V., Nandam L.S., Vaidya V.A., Bartlett P.F. (2010). Norepinephrine directly activates adult hippocampal precursors via β3-adrenergic receptors. J. Neurosci..

[bib25] Justice N.J. (2018). The relationship between stress and Alzheimer’s disease. Neurobiol. Stress.

[bib26] Justice N.J., Huang L., Tian J.Bin, Cole A., Pruski M., Hunt A.J., Flores R., Zhu M.X., Arenkiel B.R., Zheng H. (2015). Posttraumatic stress disorder-like induction elevates β-amyloid levels, which directly activates corticotropin-releasing factor neurons to exacerbate stress responses. J. Neurosci..

[bib27] Klimova B., Maresova P., Valis M., Hort J., Kuca K. (2015). Alzheimer’s disease and language impairments: social intervention and medical treatment. Clin. Interv. Aging (Vol. 10)..

[bib28] Lanté F., Chafai M., Raymond E.F., Salgueiro Pereira A.R., Mouska X., Kootar S., Barik J., Bethus I., Marie H. (2015). Subchronic glucocorticoid receptor inhibition rescues early episodic memory and synaptic plasticity deficits in a mouse model of Alzheimer’s disease. Neuropsychopharmacology.

[bib29] Lassen-Greene C.L., Steward K., Okonkwo O., Porter E., Crowe M., Vance D.E., Griffith H.R., Ball K., Marson D.C., Wadley V.G. (2017). Mild cognitive impairment and changes in everyday function over time: the importance of evaluating both speed and accuracy. J. Geriatr. Psychiatry Neurol..

[bib30] Lesuis S.L., Weggen S., Baches S., Lucassen P.J., Krugers H.J. (2018). Targeting glucocorticoid receptors prevents the effects of early life stress on amyloid pathology and cognitive performance in APP/PS1 mice. Transl. Psychiatry.

[bib31] Li D., Huang Y., Cheng B., Su J., Zhou W.X., Zhang Y.X. (2016). Streptozotocin induces mild cognitive impairment at appropriate doses in mice as determined by long-term potentiation and the morris water maze. J. Alzheimer’S. Dis..

[bib32] Mather M., Clewett D., Sakaki M., Harley C.W. (2016). Norepinephrine ignites local hotspots of neuronal excitation: how arousal amplifies selectivity in perception and memory. Behav. Brain Sci..

[bib33] Meng L., Sun Y., Zhao X., Rasmussen M., Al-Tarshan Y., Meng D.M., Liu Z., Adams D.C., McDonagh D.L. (2024). Noradrenaline-induced changes in cerebral blood flow in health, traumatic brain injury and critical illness: a systematic review with meta-analysis. Anaesthesia.

[bib34] Mishra P.K., Kahle E.H., Bettendorf A.F., Dailey J.W., Jobe P.C. (1993). Anticonvulsant effects of intracerebroventricularly administered norepinephrine are potentiated in the presence of monoamine oxidase inhibition in severe seizure genetically epilepsy-prone rats (GEPR-9s). Life Sci..

[bib35] Morilak D.A., Barrera G., Echevarria D.J., Garcia A.S., Hernandez A., Ma S., Petre C.O. (2005). Role of brain norepinephrine in the behavioral response to stress. Prog. NeuroPsychopharmacol. Biol. Psychiatry.

[bib36] Morris L.S., McCall J.G., Charney D.S., Murrough J.W. (2020). The role of the locus coeruleus in the generation of pathological anxiety. Brain Neurosci. Adv..

[bib37] Morris R. (1984). Developments of a water-maze procedure for studying spatial learning in the rat. J. Neurosci. Methods.

[bib38] Morterá P., Herculano-Houzel S. (2012). Age-related neuronal loss in the rat brain starts at the end of adolescence. Front. Neuroanat. Oct. 2012.

[bib39] Ouanes S., Popp J. (2019). High cortisol and the risk of dementia and alzheimer’s disease: a review of the literature. Front. Aging Neurosci..

[bib40] Ozdemir-Kumral Z.N., Akgün T., Haşim C., Ulusoy E., Kalpakçıoğlu M.K., Yüksel M.F., Okumuş T., Us Z., Akakın D., Yüksel M., Gören Z., Yeğen B. (2024). Intracerebroventricular administration of the exercise hormone irisin or acute strenuous exercise alleviates epileptic seizure-induced neuroinflammation and improves memory dysfunction in rats. BMC Neurosci..

[bib41] Paxinos G., Watson C. (2014).

[bib42] Popa-Wagner A., Fischer B., Platt D., Schmoll H., Kessler C. (2000). Delayed and blunted induction of mRNA for tissue plasminogen activator in the brain of old rats following pentylenetetrazole-induced seizure activity. J. Gerontol. Ser. A Biol. Sci. Med. Sci..

[bib43] Ross J.A., Van Bockstaele E.J. (2021). The Locus Coeruleus- Norepinephrine System in stress and arousal: unraveling historical, current, and future perspectives. Front. Psychiatry.

[bib44] Rostami F., Javan M., Moghimi A., Haddad-Mashadrizeh A., Fereidoni M. (2017). Streptozotocin-induced hippocampal astrogliosis and insulin signaling malfunction as experimental scales for subclinical sporadic Alzheimer model. Life Sci..

[bib45] Rostami F., Javan M., Moghimi A., Haddad-Mashadrizeh A., Fereidoni M. (2020). Prenatal stress promotes icv-STZ-induced sporadic Alzheimer’s pathology through central insulin signaling change. Life Sci..

[bib46] Salkovic-Petrisic M., Knezovic A., Hoyer S., Riederer P. (2013). What have we learned from the streptozotocin-induced animal model of sporadic Alzheimer’s disease, about the therapeutic strategies in Alzheimer’s research. J. Neural Transm..

[bib47] Schwarz L.A., Luo L. (2015). Organization of the locus coeruleus-norepinephrine system. Curr. Biol..

[bib48] Seshadri S., Wolf P.A., Beiser A., Au R., McNulty K., White R., D’Agostino R.B. (1997). Lifetime risk of dementia and Alzheimer’s disease: The impact of mortality on risk estimates in the Framingham Study. Neurology.

[bib49] Shoham S., Bejar C., Kovalev E., Weinstock M. (2003). Intracerebroventricular injection of streptozotocin causes neurotoxicity to myelin that contributes to spatial memory deficits in rats. Exp. Neurol..

[bib50] Sotiropoulos I., Catania C., Pinto L.G., Silva R., Pollerberg G.E., Takashima A., Sousa N., Almeida O.F.X. (2011). Stress acts cumulatively to precipitate Alzheimer’s disease-like tau pathology and cognitive deficits. J. Neurosci..

[bib51] Spear L.P. (2000). The adolescent brain and age-related behavioral manifestations. Neurosci. Biobehav. Rev..

[bib52] Spear L.P. (2013). Adolescent neurodevelopment. J. Adolesc. Health.

[bib53] Tahami Monfared A.A., Byrnes M.J., White L.A., Zhang Q. (2022). Alzheimer’s disease: epidemiology and clinical progression. Neurol. Ther..

[bib54] Tangen G.G., Engedal K., Bergland A., Moger T.A., Hansson O., Mengshoel A.M. (2015). Spatial navigation measured by the Floor Maze Test in patients with subjective cognitive impairment, mild cognitive impairment, and mild Alzheimer’s disease. Int. Psychogeriatr..

[bib55] Tarawneh R., Holtzman D.M. (2012). The clinical problem of symptomatic Alzheimer disease and mild cognitive impairment. Cold Spring Harb. Perspect. Med..

[bib56] Tatomir A., Micu C., Crivii C. (2014). The impact of stress and glucocorticoids on memory. Clujul Med..

[bib57] Udeh-Momoh C.T., Su B., Evans S., Zheng B., Sindi S., Tzoulaki I., Perneczky R., Middleton L.T. (2019). Cortisol, Amyloid-β, and reserve predicts Alzheimer’s disease progression for cognitively normal older adults. J. Alzheimer’S. Dis..

[bib58] van Rossum E.F.C., de Jong F.J., Koper J.W., Uitterlinden A.G., Prins N.D., van Dijk E.J., Koudstaal P.J., Hofman A., de Jong F.H., Lamberts S.W.J., Breteler M.M.B. (2008). Glucocorticoid receptor variant and risk of dementia and white matter lesions. Neurobiol. Aging.

[bib59] Vorhees C.V., Williams M.T. (2006). Morris water maze: procedures for assessing spatial and related forms of learning and memory. Nat. Protoc..

[bib60] Vyas A., Mitra R., Shankaranarayana Rao B.S., Chattarji S. (2002). Chronic stress induces contrasting patterns of dendritic remodeling in hippocampal and amygdaloid neurons. J. Neurosci..

[bib61] Wadley V.G., Okonkwo O., Crowe M., Ross-Meadows L.A. (2008). Mild cognitive impairment and everyday function: Evidence of reduced speed in performing instrumental activities of daily living. Am. J. Geriatr. Psychiatry.

[bib62] Wang J., Zhou Y., Li K., Li X., Guo M., Peng M. (2021). A noradrenergic lesion attenuates surgery-induced cognitive impairment in rats by suppressing neuroinflammation. Front. Mol. Neurosci..

[bib63] Wang Y., Li M., Tang J., Song M., Xu X., Xiong J., Li J., Bai Y. (2011). Glucocorticoids facilitate astrocytic amyloid-β peptide deposition by increasing the expression of APP and BACE1 and decreasing the expression of amyloid-β-degrading proteases. Endocrinology.

[bib64] Yoshida T., Nishioka H., Nakamura Y., Kondo M. (1985). Reduced noradrenaline turnover in streptozotocin-induced diabetic rats. Diabetologia.

[bib65] Zhang R., Tachibana T., Takagi T., Koutoku T., Denbow D.M., Furuse M. (2003). Centrally administered norepinephrine modifies the behavior induced by corticotropin-releasing factor in neonatal chicks. J. Neurosci. Res..

[bib66] Zhu H., Yan H., Tang N., Li X., Pang P., Li H., Chen W., Guo Y., Shu S., Cai Y., Pei L., Liu D., Luo M.H., Man H., Tian Q., Mu Y., Zhu L.Q., Lu Y. (2017). Impairments of spatial memory in an Alzheimer’s disease model via degeneration of hippocampal cholinergic synapses. Nat. Commun..

